# Treatment Patterns and Outcomes Among Patients With Advanced or Recurrent Endometrial Cancer Initiating First-Line Therapy in the United States

**DOI:** 10.36469/001c.87853

**Published:** 2023-10-27

**Authors:** Robert L. Coleman, Jamie Garside, Jean Hurteau, Joehl Nguyen, Monica Kobayashi

**Affiliations:** 1 Texas Oncology, The Woodlands, Texas, USA; 2 GSK, London, UK; 3 GSK, Waltham, Massachusetts, USA; 4 GSK, Collegeville, Pennsylvania; 5 GSK, Durham, North Carolina, USA

**Keywords:** endometrial cancer, real-world outcomes, survival, first-line therapy, chemotherapy, treatment outcomes, United States

## Abstract

**Background:** Patients with advanced or recurrent endometrial cancer (EC) typically have limited treatment options and poor long-term survival outcomes following first-line therapy. Real-world treatment patterns and survival outcomes data are limited for patients in this setting.

**Objectives:** The objective of this retrospective study was to describe real-world demographics, clinical characteristics, treatment patterns, and overall survival among patients in the United States with primary advanced or recurrent EC who initiated at least 1 line of therapy (LOT).

**Methods:** Patients with a diagnosis of primary advanced or recurrent EC in a real-world database from January 1, 2013, to July 31, 2021, were included. The date for inclusion was the date of EC diagnosis documentation; patients were indexed for treatment patterns and outcomes at the start of the first LOT and at the start of each subsequent LOT they initiated. Data were stratified by subgroups of patients who had mismatch repair deficient (dMMR) or microsatellite instability-high (MSI-H) tumors.

**Results:** A total of 1961 patients who received at least 1 LOT were included. Most patients in this cohort, and the dMMR/MSI-H subgroup, received a platinum combination as first-line treatment, with carboplatin-paclitaxel being the most common regimen. Only 53% of patients who received first-line treatment subsequently received second-line therapy. Of the patients who received at least 1 LOT, use of immunotherapy in the second-line setting was more common in the dMMR/MSI-H subgroup. Median overall survival ranged from 14.1 to 31.8 months across the 5 most frequently used first-line treatment regimens in the ≥1 LOT cohort and became shorter with each subsequent LOT.

**Discussion:** The use of platinum-based chemotherapy for first-line treatment of advanced or recurrent EC predominates in the real-world setting, despite the poor long-term survival outcomes associated with most of these regimens.

**Conclusions:** Patients with recurrent/advanced EC have a poor prognosis, highlighting the need for therapies with more durable benefits.

## BACKGROUND

Endometrial cancer (EC) is a common gynecological cancer in developed countries.^1^ In the United States (US), there were an estimated 65 950 new cases of EC and 12 550 EC-related deaths in 2022.^2^ Mortality rates for EC have increased in recent years (death rate, 5.1/100 000 women per year, based on 2016-2020 deaths) and are now approaching those for ovarian cancer (death rate, 6.3/100 000 women per year).^2,3^ Furthermore, estimates predict 15 000 deaths from EC in 2030, which would surpass mortality predictions of ovarian cancer in females.^4^ The increasing mortality rate over time highlights the need for new, effective therapies for patients with EC.^2,4,5^

The majority of patients are diagnosed with early-stage disease and have a favorable prognosis, with a 5-year survival rate of 95%.^6,7^ However, long-term outcomes are poor for those who have already progressed to metastatic EC at the time of diagnosis; 5-year survival rates drop to 69% upon regional metastasis, and 18% upon distant metastasis.^6^

Platinum-based doublet chemotherapy is considered first-line (1L) treatment for advanced or recurrent EC, with US and European treatment guidelines recommending carboplatin in combination with paclitaxel as a treatment option for patients with newly diagnosed advanced or recurrent EC.^8–11^ In clinical trials and observational studies, high response rates with platinum-based regimens in these patients are observed (40%-62%); however, long-term outcomes remain poor, with median overall survival (OS) rates between 13 and 41 months, and progression-free survival (PFS) rates between 6 and 15 months.^12–14^ Other 1L treatment options include single-agent chemotherapy, hormone therapy, targeted therapy, and immunotherapy, with immunotherapy licensed in both first- and second-line (2L).^8–10,15^ Clinical studies have found a positive correlation between mismatch repair deficient (dMMR) or microsatellite instability-high (MSI-H) status and response to anti–programmed cell death protein/ligand-1 (PD-[L]1) therapy, and therefore screening patients with EC for dMMR/MSI-H is recommended.^8,9^

Data are lacking on 1L treatment patterns and outcomes for patients treated for advanced or recurrent EC in the real-world setting. The objective of the current study was to describe the demographics, clinical characteristics, treatment patterns, and OS outcomes among patients with primary advanced or recurrent EC in the US who initiated at least 1 line of therapy (LOT) from a real-world database. This study was intended to support ongoing Phase 3 randomized trials of anti–PD-1 antibodies in combination with carboplatin plus paclitaxel in patients with newly diagnosed advanced or recurrent EC, by assessing whether carboplatin plus paclitaxel was representative of the standard of care in 1L therapy in the real-world setting in the US.^16,17^

## METHODS

### Study Design and Analysis Database

This was a retrospective study of patients with primary advanced or recurrent EC using the nationwide Flatiron Health electronic health record (EHR)–derived de-identified database. The Flatiron Health database is a longitudinal database, comprising de-identified patient-level structured and unstructured data, curated via technology-enabled abstraction.^18^ During the study period, the de-identified data originated from approximately 280 cancer clinics (~800 sites of care). Most patients in the database originate from community oncology settings; relative community/academic proportions may vary depending on the study cohort. Additional details on the Flatiron Health database are described in the **Supplementary Methods**.

The date for inclusion in the study was the date of advanced or recurrent EC diagnosis documentation (**Supplementary Figure S1**). Patients were indexed for follow-up of treatment characteristics and outcomes at the start date of each respective oncologist-defined, rule-based LOT. Eligible patients were followed for treatment characteristics and outcomes from the start date of the first LOT (LOT1) and at the start of each subsequent LOT that they initiated until the earliest of their last visit, a record of death, last date of structured or unstructured activity, or end date of data availability on January 31, 2022. Patients who were alive were censored on the date of the last structured or unstructured confirmed activity in the Flatiron Health database, or the latest of LOT end date.

### Objectives

The primary objectives of this study were to describe patient baseline demographic and clinical characteristics including age, race, geographic region, histology, stage of disease, MSI and MMR status, Eastern Cooperative Oncology Group performance status, and prior surgery/radiation as well as the treatment patterns and characteristics in patients initiating 1L therapy and subsequent lines of treatment, by LOT. The secondary objectives were to assess baseline characteristics among patients with shorter time to next treatment (TTNT) or death, and patients with longer TTNT or death (as a proxy measure for disease progression) in the cohort of patients who received at least 1 line of therapy (≥1 LOT cohort); to describe treatment patterns and characteristics by the top 5 most frequent 1L regimens; and to evaluate OS by the top 5 most frequent 1L treatment regimens and by LOT among patients who received ≥1 LOT.

### Study Population

Eligibility criteria were based on the key inclusion criteria from ongoing Phase 3 trials investigating 1L PD-1/PD-L1 inhibitors in combination with chemotherapy in advanced or recurrent EC.^16,17,19^

The study population included all patients with a diagnosis of primary advanced or recurrent EC in the Flatiron Health database from January 1, 2013, to July 31, 2021, defined by Flatiron Health as the advanced EC analytic cohort.

Eligible patients in the Flatiron advanced EC analytic cohort were adults (≥18 years of age as of the EC diagnosis date) with a documented diagnosis of EC (based on 2 diagnosis codes, *International Classification of Diseases* [ICD]: ICD-9 182.0 [malignant neoplasm of corpus uteri except isthmus] or ICD-10 C54.1 [malignant neoplasm of endometrium]) on or after January 1, 2013, based on structured data; at least 2 documented clinical encounters on or after January 1, 2013; and with an initial diagnosis of Stage III or Stage IV EC on or after January 1, 2013, or a diagnosis of Stage I or Stage II EC with subsequent locoregional or distant recurrence on or after January 1, 2013. Additional eligibility criteria applied to this study include an advanced or recurrent diagnosis during the period from January 1, 2013, to July 31, 2021, and at least 1 drug episode date designated as 1L in the advanced setting (except primary objective 1: baseline demographic and clinical characteristics of patients).

Patients were excluded from the Flatiron Health advanced EC analytic cohort if they had an initial diagnosis of Stage I or Stage II EC without locoregional recurrence or distant recurrence, histology consistent with uterine sarcoma (other than carcinosarcoma), or a lack of relevant unstructured documents in the Flatiron Health database for review by the abstraction team. Additional exclusion criteria applied to this study included incomplete death information (eg, missing month of death), no structured activity within 90 days after advanced EC diagnosis, or having received a clinical study drug at any time during the study period.

The overall cohort included patients who met all eligibility criteria. The ≥1 LOT cohort included all patients within the overall cohort who experienced at least 1 drug episode date designated as 1L. Patients within the ≥1 LOT cohort who had dMMR or MSI-H tumors were included in the dMMR/MSI-H subgroup.

### Endpoints

The primary endpoints were the identification of treatment patterns and characteristics (duration on therapy and TTNT) for LOT1 and LOT2. For patients in 1L, these endpoints were also described for patient subgroups receiving the top 5 most frequent drug classes and treatment regimens. Kaplan-Meier estimates for the median (interquartile range [IQR]) duration on therapy in months and median (IQR) TTNT in months were reported. A post hoc analysis was performed to determine the duration of follow-up time.

Overall survival, as a secondary endpoint, was reported by LOT1, LOT2, and LOT3, and for the top 5 most frequent regimens in LOT1. Baseline characteristics were assessed among patients who had an early progression event and those who did not, using initiation of a subsequent LOT or death as proxies for progression. The cutoff for early progressors was derived from median TTNT observed; median TTNT was defined as the period from the first drug episode date in a prior line (1L and 2L) until the earliest start date of the subsequent line (2L and third-line, respectively), death, or the date of last structured or unstructured confirmed activity in the Flatiron Health database.

Baseline characteristics and treatment patterns data were stratified by subgroups of patients who had dMMR or MSI-H tumors to examine differences in treatment patterns and characteristics.

### Statistical Analysis

All patients who met the inclusion and exclusion criteria, as described previously, were included. Analyses were conducted using SAS 9.4 or its latest version (SAS Institute, Cary, North Carolina, USA), and all statistics were descriptive. Means and medians were reported for continuous variables and counts and percentages for categorical variables; time-to-event analyses were performed using Kaplan-Meier methods.

## RESULTS

### Study Population Baseline Demographics and Clinical Characteristics

**Figure 1.**
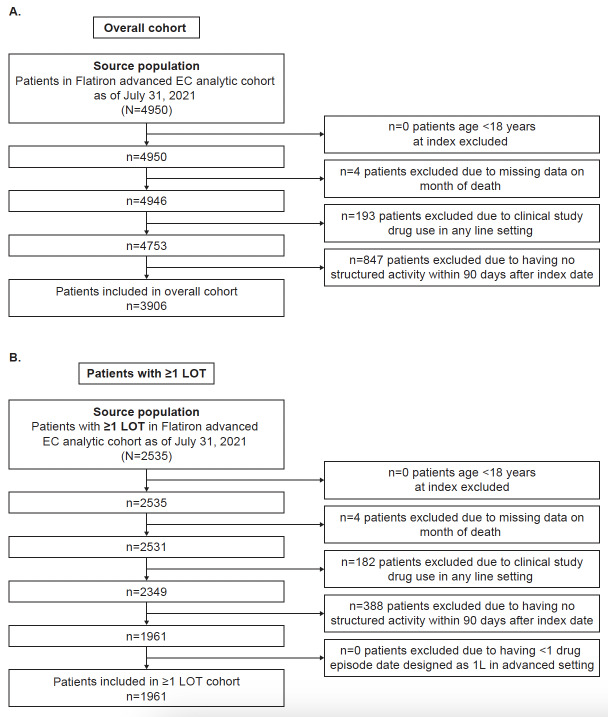
Attrition in (**A**) Overall Cohort and (**B**) ≥1 LOT Cohort Abbreviations: EC, endometrial cancer; LOT, line of therapy.

A total of 3906 and 1961 patients were included in the overall and ≥1 LOT cohorts, respectively (**Figure 1**). A total of 1961 patients in the overall cohort initiated 1L therapy and were eligible for analysis of treatment patterns and outcomes after 1L initiation (≥1 LOT cohort) (**Table 1**). In the ≥1 LOT cohort, the mean age was 66 years; most patients had advanced EC at diagnosis (1192/1961; 61%), were white (1198/1961; 61%), and had documented surgery for primary treatment of EC on or before the LOT1 index date (1098/1961; 56%). Approximately half of patients had endometrioid carcinoma (968/1961; 49%).

**Table 1.** Baseline Demographics and Patient Characteristics of the ≥1 LOT Cohort

	**≥1 LOT Cohort (n=1961)**
Age (y), mean (SD)	66 (10)
Age group (y), n (%)	
18–64	754 (38)
65–74	794 (40)
≥75	413 (21)
Year of 1L start, n (%)	
2013	102 (5)
2014	178 (9)
2015	215 (11)
2016	247 (13)
2017	279 (14)
2018	271 (14)
2019	260 (13)
2020	250 (13)
2021–2022	159 (8)
Duration of follow-up from start of 1L (mo)	
Mean (SD)	20.4 (20.9)
Median (Q1, Q3)	13 (5, 30)
Race, n (%)	
Asian	37 (2)
Black or African American	328 (17)
White	1198 (61)
Other	241 (12)
Unknown/missing	157 (8)
US Census region, n (%)	
Midwest	276 (14)
Northeast	240 (12)
South	794 (40)
West	206 (11)
Unknown/missing	445 (23)
Disease histology group, n (%)	
Endometrioid	968 (49)
Non-endometrioid	993 (51)
FIGO stage at EC diagnosis, n (%)	
I	566 (29)
II	87 (4)
III	386 (20)
IV	806 (41)
Unknown/missing	116 (6)
Baseline MSI and MMR status, n (%)	
dMMR or MSI-H	60 (3)
pMMR or MSS	128 (7)
Unknown/not tested	1773 (90)
ECOG performance status, n (%)	
0	235 (12)
1	143 (7)
2-4	29 (1)
Unknown/missing	1554 (79)
Received surgery for primary treatment of EC on or before LOT1 index date, n (%)	
Yes	1098 (56)
No	73 (4)
Missing/undocumented	790 (40)
Received radiation for primary treatment of EC on or before LOT1 index date, n (%)	
Yes	578 (30)
No	520 (27)
Missing/undocumented	863 (44)

Abbreviations: 1L, first-line; dMMR, mismatch repair deficient; EC, endometrial cancer; ECOG, Eastern Cooperative Oncology Group; FIGO, International Federation of Gynecology and Obstetrics; LOT, line of therapy; MMR, mismatch repair; MSI, microsatellite instability; MSI-H; microsatellite instability-high; MSS, microsatellite stability biomarker; pMMR, mismatch repair proficient; Q1, first quartile; Q3, third quartile.

Baseline patient characteristics were broadly similar between the overall cohort, the ≥1 LOT cohort, and the dMMR/MSI-H subgroup, with a few exceptions, including histology, stage at diagnosis, and treatments (**Supplementary Table S1**). More patients in the ≥1 LOT dMMR/MSI-H subgroup had endometrioid histology (78/81 [96%] vs 968/1961 [49%]), had Stage I disease at diagnosis (67/81 [83%] vs 566/1961 [29%]), received surgery before LOT1 (79/81 [98%] vs 1098/1961 [56%]), and had not received radiation treatment (37/81 [46%] vs 520/1961 [27%]), compared with the ≥1 LOT cohort. Baseline characteristics among patients with shorter/longer TTNT or death are shown in **Supplementary Table S2**.

### Treatment Patterns and Characteristics in 1L and 2L by LOT

**Supplementary Figure S2** shows treatment characteristics for 1L and 2L treatment by LOT regimen in the ≥1 LOT cohort and the ≥1 LOT dMMR/MSI-H subgroup. Definition 1 considers only National Comprehensive Cancer Network® (NCCN®) treatment guideline–recommended medications, regardless of which line they are recommended for. Definition 2 includes any medication regimen that contains an antineoplastic medication outside of NCCN treatment guideline recommendations for the treatment of advanced or recurrent EC in the “All other chemotherapy” group. Most patients in the ≥1 LOT cohort and in the ≥1 LOT dMMR/MSI-H subgroup received a platinum combination in 1L (1396/1961 [71%] and 98/166 [59%], respectively). Immunotherapy use was more common in the 2L setting vs 1L, and across both settings was more frequently used in the ≥1 LOT dMMR/MSI-H subgroup than in the ≥1 LOT cohort. Overall, 4% (n=74/1961) of patients received immunotherapy in the ≥1 LOT cohort and 18% (30/166) in the ≥1 LOT dMMR/MSI-H subgroup despite not being approved in the 1L setting. On-label use of immunotherapy in the 2L setting was present in 15% (151/1031) of patients in the ≥1 LOT cohort and 42% (49/118) in the ≥2 LOT dMMR/MSI-H subgroup. Use of non-NCCN-recommended regimens was rare in the 1L setting (1% [19/1961] in the ≥1L cohort) but more common in the 2L setting (6% [63/1031] in the ≥2 LOT cohort).

### Treatment Patterns and Characteristics by Top 5 Most Frequent 1L Treatment Regimens in the ≥1 LOT Cohort

**Figure 2.**
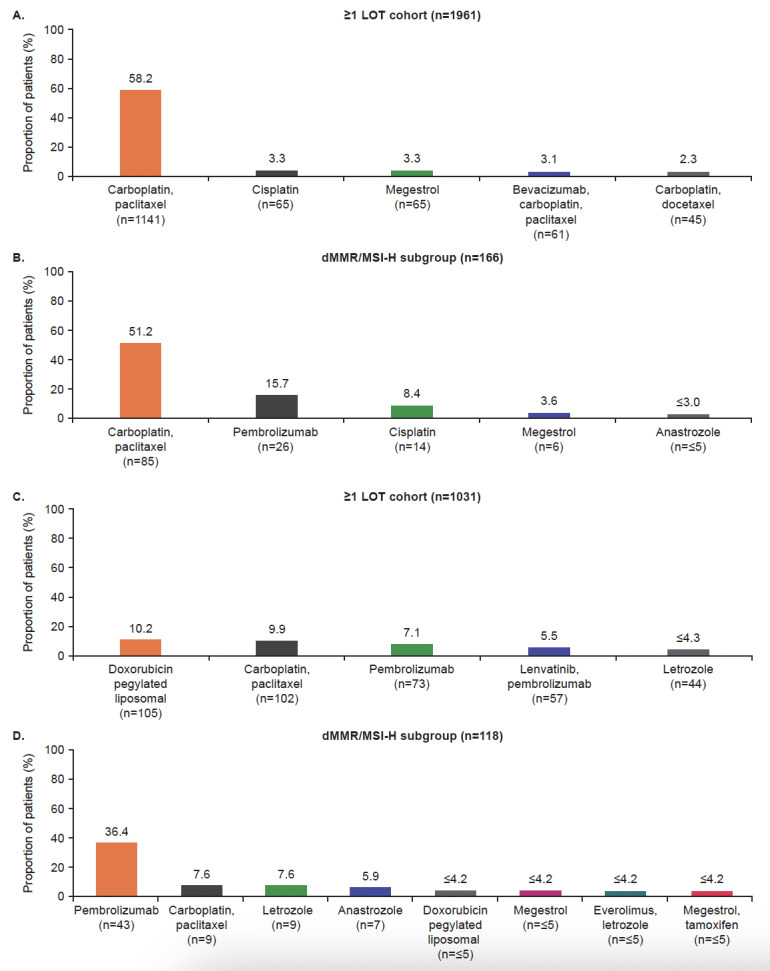
Top 5 Treatment Regimens at 1L (**A** and **B**) and 2L (**C** and **D**) More than 5 regimens are presented for the calculation for the dMMR/MSI-H subgroup in panel D. This is due to the equal percentage of patients treated with doxorubicin pegylated liposomal and megestrol (joint fourth regimens) and everolimus plus letrozole and megestrol plus tamoxifen (joint fifth regimens). Abbreviations: 1L, first-line; 2L, second-line; dMMR, mismatch repair deficient; LOT, line of therapy; MSI-H; microsatellite instability-high.

The top 5 1L and 2L treatment regimens in the ≥1 LOT cohort and dMMR/MSI-H subgroups are shown in **Figure 2**. Carboplatin plus paclitaxel was the most frequently used regimen in the 1L setting for both cohorts (58% [1141/1961] for the ≥1 LOT cohort and 51% [85/166] in the dMMR/MSI-H subgroup). Pembrolizumab was one of the most common regimens for the dMMR/MSI-H subgroup in both the 1L and 2L settings (16% [26/166] and 36% [43/118], respectively).

The median duration of therapy was similar for 1L (3.4 months) and 2L patients (3.2 months) and numerically higher for the ≥2 LOT dMMR/MSI-H subgroup than the ≥2 LOT cohort in the 2L setting (4.3 vs 3.2 months, respectively), whereas median duration of therapy was similar for both cohorts in the 1L setting (**Supplementary Table S3**). TTNT was numerically higher in 1L compared with the 2L setting overall in the ≥1 LOT cohort (10.6 vs 8.7 months, respectively); however, in the ≥1 LOT dMMR/MSI-H subgroup, TTNT was similar across 1L and 2L settings (14.9 vs 15.9 months, respectively). The median duration of treatment and TTNT for the top 5 1L regimens are shown in **Supplementary Table S4**. The duration of therapy varied from 1.0 month with cisplatin to 4.6 months with megestrol, and TTNT ranged from 9.2 months with carboplatin plus docetaxel to 19.1 months with megestrol.

### Overall Survival

**Figure 3.**
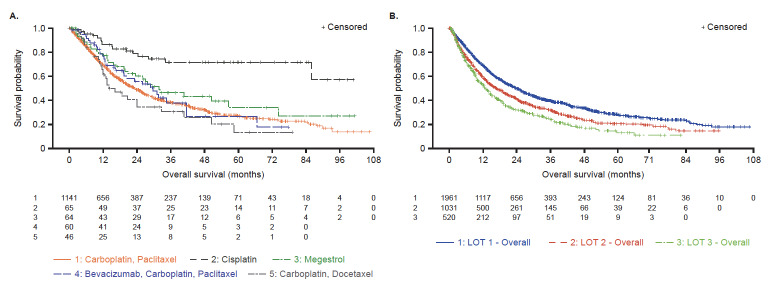
Overall Survival in ≥1 LOT Cohort by (**A**) Top 5 1L Regimens and (**B**) by LOT The top 5 1L regimens included cisplatin; however, as the median OS for cisplatin (N=65) was not reported, only the top 4 regimens are included in panel A. **Panel A**: IQR for carboplatin plus paclitaxel, 9.5-67.7 months; IQR for cisplatin, 28.1 months -NR; IQR for megestrol, 13.4 months -NR; IQR for bevacizumab plus carboplatin plus paclitaxel, 12.3-66.6 months; IQR for carboplatin plus docetaxel, 6.4-50.6 months. **Panel B**: IQR for LOT1, 9.2-73.0 months; IQR for LOT2, 6.4-46.6 months; IQR for LOT3, 5.4-35.5 months. Abbreviations: 1L, first-line; IQR, interquartile range; LOT, line of therapy; NR, not reported; OS, overall survival.

Across the top 5 1L regimens in the ≥1 LOT cohort, median OS ranged from 14.1 months with carboplatin plus docetaxel to 31.8 months with megestrol; median OS for cisplatin was not reached (**Figure 3A**). OS became shorter with each subsequent LOT (23.8 to 12.9 months in 1L and third-line, respectively) (**Figure 3B**). A marked loss in the number of patients from LOT1 (n=1961) to LOT2 (n=1031) and from LOT2 (n=1031) to LOT3 (n=520) was also observed, indicating that only 27% of patients who initiated LOT1 progressed to LOT3.

## DISCUSSION

The current study describes demographics, clinical characteristics, treatment patterns, and OS outcomes among patients in the US Flatiron Health database with primary advanced or recurrent EC who initiated at least 1 LOT. Data for a subgroup of patients with dMMR/MSI-H tumors were also analyzed to compare the treatment patterns and outcomes. Most patients in the ≥1 LOT cohort in the present study had received surgery and/or radiation therapy before LOT1 and already had advanced EC when diagnosed, with a high proportion of patients (41%) with Stage IV EC at diagnosis. Our findings indicate that standard of care treatments for patients with primary advanced or recurrent EC in the real world continue to be chemotherapy-based, despite poor long-term survival outcomes of most chemotherapy regimens.^5,12–14^ Combination therapy with carboplatin plus paclitaxel was the most common 1L regimen, in line with guideline recommendations,^8–10^ in both the overall cohort and the dMMR/MSI-H subgroup. Pembrolizumab, a PD-1 inhibitor, was one of the most common regimens used for patients with dMMR/MSI-H EC in both 1L and 2L settings. Pembrolizumab was used as 1L therapy in approximately 16% of patients in this subgroup, which may reflect potential inclusion of patients in clinical trials, given this immunotherapy is not approved for 1L use.^20^

The median duration of therapy was similar for patients treated in 1L and 2L in the ≥1 LOT cohort, while being slightly numerically higher in the dMMR/MSI-H subgroup in the 2L setting; this may be due to the greater use of immunotherapy, and lower use of chemotherapy, within this subgroup. TTNT was 10.6 and 8.7 months in the ≥1 LOT cohort, for 1L and 2L, respectively. In real-world studies, TTNT has been considered a proxy for progression.^21,22^ In this study, TTNT was similar to the PFS rates of 8 to 14 months observed in recent clinical studies in patients with advanced or recurrent EC.^12,23^ The short duration of treatment in 1L reflects that platinum-based chemotherapy is generally administered for a fixed duration of 6 to 7 cycles, each of which is typically 21 days.^10,24^ The short duration of treatment in 2L compared with TTNT may suggest that 2L treatment is being administered in fixed cycles, rather than until disease progression; however, this could be an artifact of the real-world setting, and, therefore, any interpretation should be made with caution.

Other studies evaluating real-world outcomes in patients with advanced EC include a retrospective cohort analysis by Monk et al, which also used data from the Flatiron Health database in the US. The authors reported a median (IQR) duration of therapy of 9.2 (4.1-23.2) months in the 1L setting and 5.9 (2.8-12.4) months in the 2L setting.^25^ Similarly, Liu et al reported a median 1L treatment duration of 9.5 months and 2L treatment duration of 7.6 months in patients with advanced or recurrent EC in the US who had received platinum-based chemotherapy, followed by 2L antineoplastic therapy.^26^ The durations of therapy for both 1L and 2L settings reported in these studies are higher than those reported in the current study in both the ≥1 LOT and dMMR/MSI-H cohorts. This could be explained by disparities in the proportions of Stage III (20% vs 49%) and Stage IV (40% vs 25%) between this study and Monk et al, respectively.^25^ Further, Liu et al defined duration of LOT as including both the treatment and treatment-free interval.^26^

In the present study, the median OS for the overall cohort decreased with subsequent LOT. The highest OS was observed in patients treated with hormone therapy; this may in part be due to the patients being selected for this treatment regimen typically including those with low-grade, hormone receptor–positive tumors, who tend to have a better prognosis than patients with hormone receptor–negative tumors.^9,10,27^ Median OS with the most frequent 1L regimen, carboplatin plus paclitaxel, was 23.3 months in the ≥1 LOT cohort, which is lower than that reported in a clinical trial (GOG0209) of carboplatin plus paclitaxel (37 months)^12^ and that reported by Monk et al in patients with advanced or recurrent EC (49.6 months).^25,28^ Differences in OS between Monk et al and the present study may be due to different patient indexing; this study indexed patients upon systemic 1L treatment initiation, whereas Monk et al included all patients and thus may have captured those patients cured by surgery and/or radiotherapy who did not require systemic treatment.^25^ In the present study, we restricted our analyses to only those patients who received systemic 1L treatment to eliminate additional variation that could not be adequately accounted for or controlled. Furthermore, the disparities in proportions of Stage III and Stage IV patients between this study and GOG0209 (Stage III 20% vs 41.7% and Stage IV 41% vs 31%, respectively) and the previously mentioned disparities between this study and Monk et al may account for the observed differences in OS.^12,25^

Duration of therapy and OS observed with cisplatin monotherapy in this study were shorter (1 month) and longer (not reached), respectively, than would typically be expected with this treatment and compared with observations across other treatments. These outcomes are influenced by patient baseline demographics, such as prior therapy, age, and performance status. These demographics were not stratified by 1L treatment regimens but may account for the observed variation, resulting in potential discrepancies between real-world and clinical trial data.

A loss in the number of patients from LOT1 to LOT2 and from LOT2 to LOT3 was observed in the present study; this may be reflective of the poor long-term outcomes for patients with advanced EC in the real world^21,29^ and the limited efficacy of therapeutic options available during the study.^12–14^ With the ongoing development of improved treatment regimens, the loss in patients between LOTs may decrease in the future.

Assessing characteristics of real-world patients and treatment patterns helps to contextualize clinical trial data and their populations. In particular, this study provides perspective for ongoing Phase 3 trials of anti–PD-(L)1 inhibitors in combination with platinum-based chemotherapy in the 1L setting of advanced or recurrent EC.^16,17,19^ These trials include RUBY (NCT03981796) and NRG-GY018 (NCT03914612), which demonstrated improved survival outcomes of anti-PD-(L)1 inhibitors in combination with standard of care carboplatin plus paclitaxel vs standard of care alone,^30,31^ and the ongoing AtTEnd trial (NCT03603184).^17^ By aligning eligibility criteria to the real-world cohort, this study provides information on typical real-world treatment patterns and OS outcomes relevant to the trials of anti–PD-(L)1 inhibitors in combination with platinum-based chemotherapy.^16,17,19^ Until the recent publication of data from the RUBY and NRG-GY018 studies in March 2023,^30,31^ carboplatin-paclitaxel was considered the 1L standard of care chemotherapy based on data published from the GOG0209 study in 2012, which demonstrated that carboplatin-paclitaxel was noninferior to doxorubicin-cisplatin-paclitaxel with a more favorable toxicity profile.^32–34^ The results from the present study confirm carboplatin plus paclitaxel as the 1L standard of care treatment most widely used in the real-world US setting (study period 2013-2022), validating the choice of control arm treatment regimens in the ongoing Phase 3 studies.^16,17,19,35^

Limitations of the present study include the fact that results were limited to the study population from the Flatiron Health database, defined as the advanced EC analytic cohort, which may not be fully reflective of the EC patient population in the US. These findings may not apply to global populations because patient demographics, standards of care, and healthcare delivery systems may vary globally. Similarly, because most patients in the overall cohort were treated within community centers, these real-world outcomes may not be fully generalizable to patients receiving care in academic settings. In addition, the Flatiron Health data are derived from EHRs, which are used for clinical practice management and not primarily for research, so there are some inherent limitations in applying the data to analytic review.^36^ The level of detail available was also limited by the information reported in a patient’s EHR; data for some parameters including ECOG performance status, receipt of surgery or radiation, and MSI/MMR status were not available for a significant number of patients. While the Society for Gynecologic Oncology has recommended MSI/MMR testing in the US to screen patients with endometrial cancer for Lynch syndrome since 2014,^37^ this guidance may not have been consistently applied, which could have resulted in the high proportion of missing data observed for MSI/MMR status in the present study. In addition, it is not always possible to collect informative clinical characteristics from EHRs. For example, a limitation of the Flatiron Health EHR-derived data is that they are collected only in oncology practices, so other comorbidities captured in primary care may be missing, and, as a result, comorbidity index could not be calculated. However, the composite mortality endpoint of Flatiron Health has been shown to have high sensitivity in other tumor types, representing a strength of this study.^38,39^ Other limitations included the fact that treatment and OS outcomes were described among various patient subgroups but not directly compared, with no adjustment made for confounding variables in these analyses. Data were not stratified by surgical outcome, which may impact on overall prognosis. Furthermore, misclassification of treatment and outcomes resulting from an absence of clinical care information in settings outside the oncology clinic may have occurred. The use of predefined LOTs limited the ability to redefine LOTs, including other treatment strategies that may be employed in 1L treatment.

## CONCLUSIONS

Overall, use of platinum-based therapies, including the standard of care treatment carboplatin plus paclitaxel, predominated in 1L in the ≥1 LOT cohort and in the dMMR/MSI-H subgroup, likely due to the well-established clinical benefit of this combination therapy. This study shows that, when initiated at 1L, platinum-based regimens are the most frequently used treatment for patients with advanced or recurrent EC, despite the limited long-term survival outcomes associated with these therapies and the short duration of response, as represented by TTNT, a surrogate of PFS. The median OS for patients in LOT1 was less than 2 years, which highlights the need for new effective therapies in patients with advanced or recurrent EC. As further research into the complexities of advanced or recurrent EC is conducted, it is hoped that new and emerging therapies will enable a patient-centered treatment approach and will improve survival outcomes for these patients.

### Author contributions

R.L.C. and J.H. contributed to data analysis and interpretation, drafting the manuscript and critical revision of the paper for important intellectual content. J.G., J.N., and M.K. contributed to study conceptualization and design, data analysis and interpretation, drafting the manuscript and critical revision of the paper for important intellectual content.

### Disclosures

R.L.C. has received consulting fees, grants, and honoraria from AstraZeneca, Clovis Oncology, Janssen, and Merck; consulting fees and honoraria from Aravive, Eisai, Novocure, Oncomed/Mateo, OncoQuest, OncoSec, and Tesaro/GSK; consulting fees from AbbVie; grants and honoraria from Roche/Genentech; grants from Genmab and V-Foundation. J.G. is an employee of GSK. J.H. and M.K. are employees of GSK and hold stocks and shares in the company. J.N. was an employee of GSK at the time of the study and holds stocks and shares in the company. J.N. is an employee of Bayer.

### Data sharing

The data that support the findings of this study have been originated by Flatiron Health, Inc. These de-identified data may be made available upon request and are subject to a license agreement with Flatiron Health; interested researchers should contact DataAccess@flatiron.com to determine licensing terms. GSK makes available anonymized individual participant data and associated documents from interventional clinical studies that evaluate medicines, upon approval of proposals submitted to: https://www.gsk-studyregister.com/en/. For other types of GSK-sponsored research, study documents without patient-level data, and clinical studies not listed, please submit an inquiry through the website.

## Supplementary Material

Online Supplementary Material
