# Supplementary material for: Treatment Patterns and Outcomes Among Patients With Advanced or Recurrent Endometrial Cancer Initiating First-Line Therapy in the United States
Source: J Health Econ Outcomes Res. 2023 Oct 27;10(2):82–90. doi: 10.36469/001c.87853 (PMC10613433; doi:10.36469/001c.87853)
Supplement: Online Supplementary Material [file jheor_2023_10_2_87853_185158.pdf]

JHEOR
Journal of Health Economics and Outcomes Research

Online Supplementary Material

Treatment Patterns and Outcomes Among Patients With Advanced or Recurrent Endometrial Cancer Initiating First-Line Therapy in the United States. *JHEOR*. 2023;10(2):82-90. doi:10.36469/jheor.2023.87853

Supplementary Methods

Figure S1: Study Design

Figure S2: Treatment Characteristics for 1L and 2L Treatment by Categorical LOT Regimen in the ≥1 LOT Cohort and dMMR/MSI-H Subgroup

Table S1: Baseline Demographics and Patient Characteristics of the Overall and dMMR/MSI-H Cohorts

Table S2: Baseline Demographics and Patient Characteristics Among Patients With Shorter/Longer TTNT or Death

Table S3: Duration of Therapy and TTNT

Table S4: Duration of Therapy and TTNT by Treatment Group by Top 5 1L Treatments

This supplementary material has been provided by the authors to give readers additional information about their work.

This is an open-access article distributed under the terms of the Creative Commons Attribution 4.0 International License (CCBY-4.0). View this license's legal deed at http://creativecommons.org/licenses/by/4.0 and legal code at http://creativecommons.org/licenses/by/4.0/legalcode for more information.

SUPPLEMENTARY METHODS

Data Collection

- The Flatiron Health database is a longitudinal database, comprising de-identified patient-level structured and unstructured data, curated via technology-enabled abstraction. The Flatiron Health database data are de-identified and subject to obligations to prevent re-identification and protect patient confidentiality. The de-identified patient-level data in the electronic health records included structured data (eg, laboratory values, prescribed drugs) in addition to unstructured data collected via technology-enabled chart abstraction from physicians notes and other unstructured documents (eg, biomarker reports).
- The Flatiron Health database contained patient demographics, such as birth year, sex, race/ethnicity, state, and information on the primary physician. Diagnosis information included date of diagnosis (identified using *International Classification of Diseases, Ninth Revision [ICD-9] and International Classification of Diseases, Tenth Revision* [ICD-10] codes, and diagnosis description), the stage of disease at the time of diagnosis, and histology. Additional information contained in the Flatiron Health database included patients visits, laboratory information, type of insurance, and mortality data.
- Oncologist-defined, rule-based line of therapy (LOT) in the Flatiron Health database was defined as eligible drug episode plus other eligible drugs given within 28 days. When a gap in drug episode of >120 days occurred, the LOT number was advanced. The LOT start date was the initiation of first episode of an eligible drug within a line.

Figure S1. Study Design

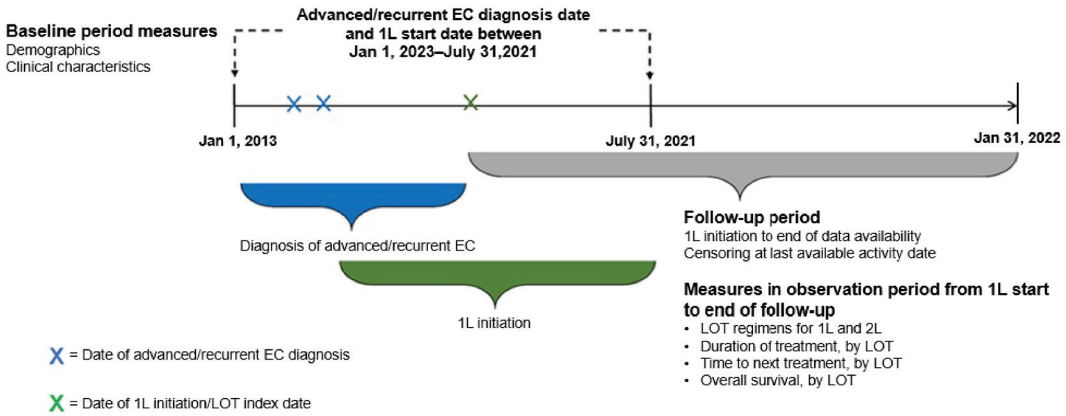

Abbreviations: 1L, first-line; 2L, second-line; EC, endometrial cancer; LOT, line of therapy.

Figure S2. Treatment Characteristics of 1L and 2L Treatment by Categorical LOT Regimen in the ≥1 LOT Cohort and dMMR/MSI-H Subgroup

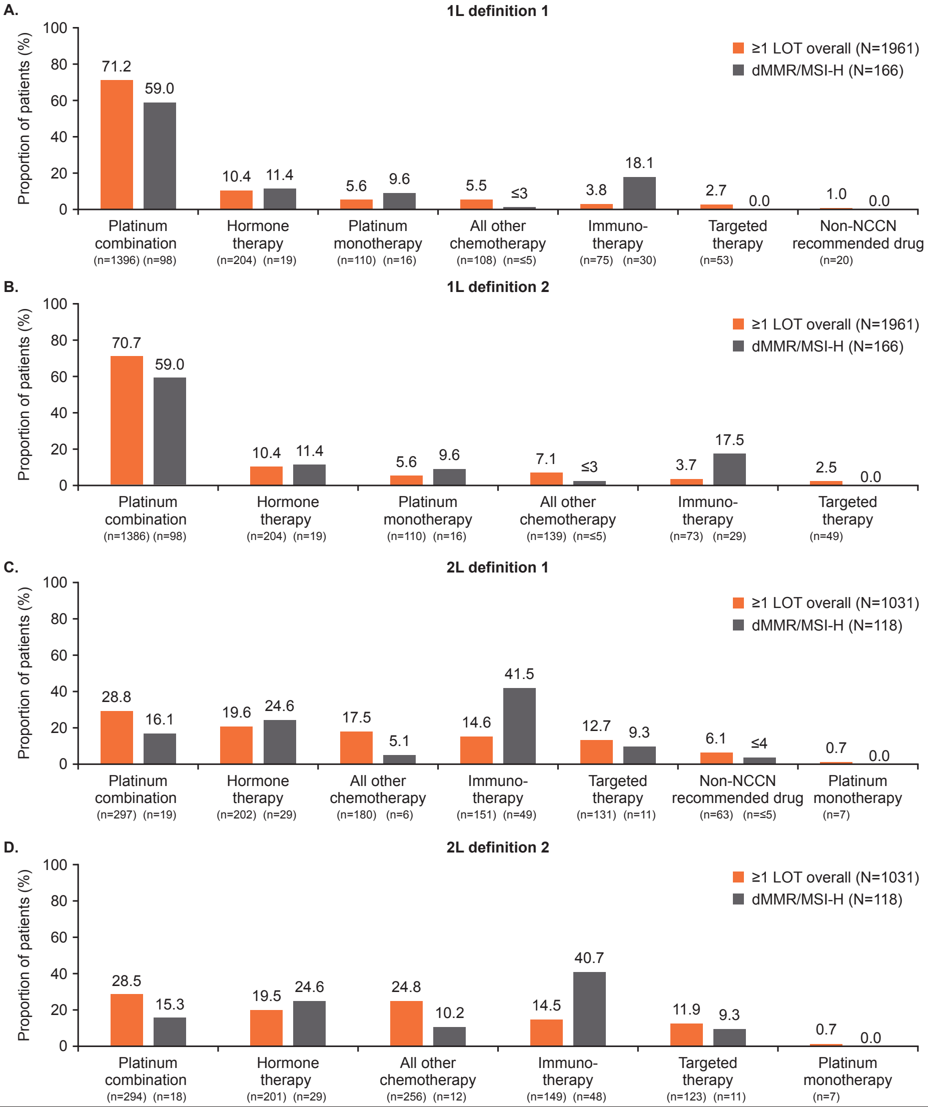

dMMR/MSI-H status was assessed based on most recent laboratory results before start of LOT 1. Definition 1 considers only treatment guideline-recommended medications regardless of which line they are recommended for. Definition 2 includes any medication regimen that contains an antineoplastic medication outside of NCCN treatment guideline recommendations for the treatment of advanced or recurrent EC in the "All other chemotherapy" group.

Abbreviations: 1L, first-line; 2L second-line; dMMR, mismatch repair deficient; EC, endometrial cancer; LOT, line of therapy; MSI-H; microsatellite instability-high; NCCN, National Comprehensive Cancer Network.

Table S1. Baseline Demographics and Patient Characteristics of the Overall and dMMR/MSI-H Cohorts

	**Overall Cohort (N=3906)**	**dMMR/MSI-H Subgroup (n=81)**
Mean age, y (SD)	65.8 (10.1)	66.4 (10.5)
Age group (y), n (%)		
18-64	1599 (41)	26 (32)
65-74	1439 (37)	37 (46)
≥75	868 (22)	18 (22)
Year of advanced/recurrent diagnosis, n (%)		
2013	344 (9)	≤5 (≤6)
2014	394 (10)	≤5 (≤6)
2015	458 (12)	≤10 (≤12)
2016	481 (12)	≤10 (≤12)
2017	539 (14)	13 (16)
2018	520 (13)	13 (16)
2019	507 (13)	22 (27)
2020	460 (12)	11 (14)
2021	203 (5)	≤5 (≤6)
Duration of follow-up from advanced/recurrent diagnosis date (mo)		
Mean (SD)	25.1 (23.8)	26.7 (20.6)
Median (Q1, Q3)	17 (7, 37)	24 (9, 40)
Race, n (%)		
Black or African American	582 (15)	12 (15)
White	2485 (64)	60 (74)
Other	513 (13)	≤10 (≤12)
Unknown/missing	326 (8)	≤10 (≤12)
US Census region, n (%)		
Midwest	550 (14)	14 (17)
Northeast	386 (10)	≤10 (≤12)
South	1644 (42)	21 (26)
West	389 (10)	≤10 (≤12)
Unknown/missing	937 (24)	32 (40)
Disease histology group, n (%)		
Endometrioid	2158 (55)	78 (96)
FIGO stage at EC diagnosis, n (%)		
I	796 (20)	67 (83)
II	124 (3)	9 (11)
III	1748 (45)	≤5 (≤6)
IV	1055 (27)	≤5 (≤6)
Unknown/missing	183 (5)	≤5 (≤6)
Baseline MSI and MMR status, n (%)		
dMMR or MSI-H	81 (2)	81 (100)
pMMR or MSS	166 (4)	0
Unknown/not tested/other	3659 (94)	0

Table S1. Baseline Demographics and Patient Characteristics of the Overall and dMMR/MSI-H Cohorts

	**Overall Cohort (N=3906)**	**dMMR/MSI-H Subgroup (n=81)**
ECOG performance status, n (%)		
0	410 (11)	16 (20)
1	220 (6)	17 (21)
2	54 (1)	≤5 (≤6)
3	≤5 (<1)	≤5 (≤6)
4	≤5 (<1)	≤5 (≤6)
Unknown/missing	3216 (82)	45 (56)
Received surgery for primary treatment of EC on or before LOT1 index date, n (%)		
Yes	2771 (71)	79 (98)
No	123 (3)	≤5 (≤6)
Missing/undocumented	1012 (26)	≤5 (≤6)
Received radiation for primary treatment of EC on or before LOT1 index date, n (%)		
Yes	1512 (39)	42 (52)
No	1259 (32)	37 (46)
Missing/undocumented	1135 (29)	≤5 (≤6)

≤5 and ≤10 reflect small sample sizes.
Abbreviations: dMMR, mismatch repair deficient; EC, endometrial cancer; ECOG, Eastern Cooperative Oncology Group; FIGO, International Federation of Gynecology and Obstetrics; LOT, line of therapy; MMR, mismatch repair; MSI, microsatellite instability; MSI-H; microsatellite instability-high; MSS, microsatellite stability biomarker; pMMR, mismatch repair proficient; Q1, first quartile; Q3, third quartile.

Table S2. Baseline Demographics and Patient Characteristics Among Patients With Shorter/Longer TTNT or Death

	**Censored <323 Days (Median = 205)**	**TTNT or Death <323 Days (Median = 1161)**	**TTNT or Death ≥323 Days (Median = 595)**
Mean age, y (SD)	66.0 (9.8)	66.5 (9.4)	65.7 (9.7)
Age group (y), n (%)			
18-64	84 (41)	425 (37)	245 (41)
65-74	72 (35)	494 (43)	228 (38)
≥75	49 (24)	242 (21)	122 (21)
Race, n (%)			
Black or African American	29 (14)	216 (19)	83 (14)
White	118 (58)	699 (60)	381 (64)
Other	33 (16)	163 (14)	82 (14)
Unknown/missing	25 (12)	83 (7)	49 (8)
US Census region, n (%)			
Midwest	20 (10)	164 (14)	92 (15)
Northeast	26 (13)	144 (12)	70 (12)
South	88 (43)	474 (41)	232 (39)
West	25 (12)	119 (10)	62 (10)
Unknown/missing	46 (22)	260 (22)	139 (23)
Disease histology group, n (%)			
Endometrioid	96 (47)	546 (47)	326 (55)
Non-endometrioid	109 (53)	615 (53)	269 (45)
FIGO stage at EC diagnosis, n (%)			
I	39 (19)	306 (26)	221 (37)
II	9 (4)	53 (5)	25 (4)
III	58 (28)	243 (21)	85 (14)
IV	85 (42)	489 (42)	232 (39)
Unknown/missing	14 (7)	70 (6)	32 (5)
Baseline MSI and MMR status, n (%)			
dMMR or MSI-H	≤10 (<5)	34 (3)	24 (4)
pMMR or MSS	≤10 (<5)	77 (7)	44 (7)
Unknown/not tested/other	196 (96)	1050 (90)	527 (89)
ECOG performance status, n (%)			
0	15 (7)	144 (12)	76 (13)
1	10 (5)	104 (9)	29 (5)
2	≤5 (≤3)	17 (2)	7 (1)
3	≤5 (≤3)	≤5 (≤1)	≤5 (≤1)
4	≤5 (≤3)	≤5 (≤1)	≤5 (≤1)
Unknown/missing	176 (86)	895 (77)	483 (81)
Received surgery for primary treatment of EC on or before LOT1 index date, n (%)			
Yes	106 (52)	643 (55)	349 (59)
No	15 (7)	41 (4)	17 (2)
Missing/undocumented	84 (41)	477 (41)	229 (39)

Table S2. Baseline Demographics and Patient Characteristics Among Patients With Shorter/Longer TTNT or Death

	**Censored <323 Days (Median = 205)**	**TTNT or Death <323 Days (Median = 1161)**	**TTNT or Death ≥323 Days (Median = 595)**
Received radiation for primary treatment of EC on or before LOT1 index date, n (%)			
Yes	59 (29)	342 (30)	177 (30)
No	47 (23)	301 (26)	172 (29)
Missing/undocumented	99 (48)	518 (45)	246 (41)

≤5 reflects small sample sizes.
Abbreviations: dMMR, mismatch repair deficient; EC, endometrial cancer; ECOG, Eastern Cooperative Oncology Group; FIGO, International Federation of Gynecology and Obstetrics; LOT, line of therapy; MMR, mismatch repair; MSI, microsatellite instability; MSI-H; microsatellite instability-high; MSS, microsatellite stability biomarker; pMMR, mismatch repair proficient; TTNT, time to next treatment.

Table S3. Duration of Therapy and TTNT

	**Median (IQR)**			
	**Duration of Therapy (mo)**		**TTNT (mo)**	
	**1L**	**2L**	**1L**	**2L**
≥1 LOT (n=1961)	3.4 (1.8–5.2)	3.2 (1.4–6.9)	10.6 (5.1–36.7)	8.7 (3.9–27.0)
dMMR/MSI-H subgroup (n=166)	3.5 (1.9–6.8)	4.3 (1.6–18.4)	14.9 (4.6–NR)	15.9 (5.4–46.3)

Abbreviations: 1L, first-line; 2L, second-line; dMMR, mismatch repair deficient; IQR, interquartile range; LOT, line of therapy; MSI-H; microsatellite instability-high; NR, not reported; TTNT, time to next treatment.

Table S4. Duration of Therapy and TTNT by Treatment Group by Top 5 1L Treatments

**Treatment Group**	**n**	**Median (IQR)**	
		**Duration of Therapy (mo)**	**TTNT (mo)**
Carboplatin + paclitaxel	1141	3.4 (2.1-4.6)	10.1 (5.3-27.8)
Cisplatin	65	1.0 (0.9-1.2)	11.9 (2.8-NR)
Megestrol	64	4.6 (2.1-18.7)	19.1 (3.9-NR)
Bevacizumab + carboplatin + paclitaxel	60	4.2 (3.0-8.9)	14.0 (7.4-26.7)
Carboplatin + docetaxel	46	3.4 (1.4-4.4)	9.2 (2.9-16.9)

Abbreviations: 1L, first-line; IQR, interquartile range; NR, not reached; TTNT, time to next treatment.
